# Effect of Clopidogrel combined with aspirin in the treatment of acute progressive cerebral infarction: A retrospective single-center analysis

**DOI:** 10.12669/pjms.40.5.9206

**Published:** 2024

**Authors:** Huiying Huang, Shan Zhang, Hong Du, Yonghua Guo, Hui Zheng

**Affiliations:** 1 Huiying Huang, Department of Neurology, People’s Hospital of Leshan, Leshan, Sichuan Province 614000, P.R. China; 2 Shan Zhang, Department of Neurology, People’s Hospital of Leshan, Leshan, Sichuan Province 614000, P.R. China; 3 Hong Du, Department of Neurology, People’s Hospital of Leshan, Leshan, Sichuan Province 614000, P.R. China; 4 Yonghua Guo, Department of Neurology, People’s Hospital of Leshan, Leshan, Sichuan Province 614000, P.R. China; 5 Hui Zheng Department of Neurology, Chengdu First People’s Hospital, 18 Wanxiang North Road, Chengdu, Sichuan Province 610095, P.R. China

**Keywords:** Acute progressive cerebral infarction, Aspirin, Clopidogrel

## Abstract

**Objective:**

To explore the effect of clopidogrel combined with aspirin in the treatment of acute progressive cerebral infarction (APCI).

**Methods:**

We retrospectively analyzed the records of 190 patients with APCI admitted to Chengdu First People’s Hospital from September 2020 to April 2023. The records were divided into an aspirin group (76 cases), a clopidogrel group (72 cases), and a clopidogrel plus aspirin group (42 cases) according to the treatment records. We compared the efficacy of the three treatment outcomes by analyzing the National Institutes of Health Stroke Scale (NIHSS) scores, and the levels of serum inflammatory factors (IL-8, TNF-α, and IL-1β), cone like protein-1 (VILIP-1), and caveolin-1 (Cav-1).

**Results:**

The total efficacy of the combination group (97.62%) was significantly higher than those of the aspirin group (73.68%) or the clopidogrel group (79.17%) (*p*<0.05). After treatment, the NIHSS scores, inflammatory factor levels, serum VILIP-1 and Cav-1 levels were significantly lower than those before treatment in the three groups, but all the levels were significantly lower in the combination group (all *p*<0.05).

**Conclusions:**

Our results indicate that compared with aspirin alone or clopidogrel alone, the combination of aspirin and clopidogrel is more effective for the treatment of APCI. The combination regimen effectively lowers serum inflammatory factors (IL-8, TNF-α, and IL-1β), as well as the VILIP-1 and Cav-1 levels.

## INTRODUCTION

Cerebral infarctions are a common cerebrovascular disease.^1^ With the increase in the number of elderly people globally, the incidence of cerebral infarction has been increasing year by year and has become a major disease that seriously threatens the life and well-being of the people.^1^,^2^ Acute progressive cerebral infarction (APCI) is characterized by sudden onset of hemiparesis, aphasia, dizziness, headache, nausea and vomiting, crooked mouth and decrease in level of consciousness, and is a subtype of cerebral infarction in which the continuous progression of neurological deficits ensues in patients within 6-7 days after onset, despite medical interventions received.^2^,^3^ APCI can cause sustained damage to a patient’s brain, leading to symptoms such as hemiplegia and abnormal speech function. APCIs cause high disability and case fatality rates; and, prompt and effective interventions are crucial.^3^,^4^

Treatment of APCI including anticoagulant, platelet anti-agglutinating agents, agents such as low molecular weight dextran, and fibrinolytic drugs, and clopidogrel and aspirin have been widely used in clinical practice.^5^-^7^ Compared with single medication, researchers believe that a combination of the two drugs mentioned above may result in a synergistic effect that should improve treatment effectiveness.^6^,^7^ Most studies have been focused on the recurrence rate and hematological adverse effects after combined treatment, but few on changes in serum inflammatory factor levels and serum levels of Visinin-like protein-1 (VILIP-1) and Caveolin-1 (Cav-1).^7^,^8^ Physicians at our hospital have been using clopidogrel combined with aspirin to treat APCIs, and we conducted a retrospective analysis of the treatment outcomes of such patients to provide a reference and suggestions for other medical personnel.

## METHODS

In this retrospective single-center study, the clinical data of 190 patients with APCI treated in Chengdu First People’s Hospital from September 2020 to April 2023 were retrospectively reviewed. The patients were assigned to the following three groups according to the treatment they received: aspirin group (n=76), clopidogrel group (n=72), and combination (aspirin with clopidogrel) group (n=42).

### Inclusion criteria:

- Patients diagnosed on the basis of APCI diagnostic criteria.^9^
- Patients with first-time onset APCI.
- Patients with treatment initiation within three days of symptoms onset.
- Patients with available cranial CT or MRI.
- Patients with complete clinical data.

### Exclusion criteria:

- Patients with organic lesions or important organ function impairments.
- Patients with major infections and/or immunological diseases.
- Patients with a history of thrombolysis or anticoagulation treatment three months before admission.
- Patients allergic to aspirin and clopidogrel.

### Ethical Approval:

We conducted all procedures involving human participants in accordance with the ethical standards of the institutional and national research committees and with the tenets of the Helsinki Declaration (as revised in 2013).

### Ethical Approval:

We obtained written informed consents from the patients or their legal guardians. The medical ethics committee of our hospital approved this study (No. 20230612-1, Date: 2023-06-12).

### Treatment methods:

All patients were given routine treatments such as blood vessel dilation, anticoagulation, brain protection, blood sugar lowering, blood lipid lowering, and blood pressure control. We administered aspirin (Huadong Medicine [Xi’an] Bohua Pharmaceutical; approval number, H61022717) in an initial dose of 0.3g/day. If the patient experienced nausea, vomiting, discomfort in the upper abdomen, or painful gastrointestinal reactions, we adjusted the dosage to 0.1g/day. For patients prescribed clopidogrel (Shenzhen Salubris Pharmaceuticals Pharmaceutical; approval number, H20120035) we administered 0.3g/day initially and adjusted the dose gradually to 0.75 mg/day according to their condition. Patients in all groups were continuously treated for 14 days.

### Relevant patient indicators before and after 14 days of treatment:

- We calculated National Institutes of Health Stroke Scale (NIHSS) scores. The scale involves 15 items scored from 0 to 42 points, with higher scores indicating more severe neurological impairments.^10^
- We measured serum inflammatory factor (Interleukin 8 [IL-8], tumor necrosis factor-α [TNF-α] and interleukin-1β [IL-1β]) levels using enzyme-linked immunosorbent assays (Shanghai Lianmai Biotechnology, Shanghai, China).
- We measured serum levels of VILIP-1 and Cav-1 with enzyme-linked immunosorbent assays (Shanghai Lianmai Biotechnology, Shanghai, China).
- We assess the treatment efficacy on the basis of NIHSS score reductions after treatment: we considered reductions ≥ 46% as indicating significant effectiveness; reductions between 18% and 45% as indicating some effectiveness; and, reductions <18% as ineffective. The total efficacy was calculated using the formulae: Total effective rate = (total number of patients - number of ineffective treatments)/total number of patients × 100%.

### Statistical Analysis:

We analyzed data using SPSS22.0. We expressed normally distributed data using means and standard deviations (*χ̅*±*S*). We initially applied single factor analyses of variance to compare variables between multiple groups; then, we used LSD tests for comparisons between two groups and paired *t*-tests for comparisons within the same group. We represented non-normal data using medians and interquartile ranges (IQRs). We used a Kruskal Wallis H test for comparisons of medians between groups, and then the Nemenyi method for comparisons between two groups. We applied the Wilcoxon symbolic rank test for comparisons within the same group. We expressed counting data as numbers and percentages (%); and, we used Chi-squared tests for comparisons between groups. According to the test levels, we considered *p-values* <0.05 as indicative of statistically significant differences.

## RESULTS

We analyzed variables in 190 cases meeting our inclusion criteria, 76 patients received aspirin alone, 72 received clopidogrel alone, and 42 received a combination of aspirin and clopidogrel. The ages ranged from 39 to 79 years with a mean of 63.49±7.54 years. The disease course ranged between 1.00 (0.5, 2) days. The body mass indexes (BMIs) of patients ranged from 18.7 kg/m² to 30.7 kg/m² with a mean of 23.67±1.80 kg/m². We found similar baseline data values among the three groups (*p*>0.05; Table-I).

**Table-I:** Comparison of baseline data among three groups.

Group	Gender (men/women)	Age (years)	BMI (kg/m²)	Course of disease (days)	Education level (years)
Aspirin group (n=76)	38/38	64.43±7.87	23.85±2.38	1 (1,1)	9 (7.5,9.5)
Clopidogrel group (n=72)	41/31	62.89±7.56	23.58±2.14	1 (1,1)	9 (8,10)
Combination group (n=42)	24/18	63.52±9.00	24.16±2.39	1 (1,1)	8 (8,10)
*χ^2^/F/Z*	0.905	0.693	0.875	1.489	0.467
*p-value*	0.636	0.501	0.419	0.475	0.792

The total efficacy of the combination group was 97.62%, significantly higher than the 73.68% of the aspirin group or the 79.17% of the clopidogrel group (all *p*<0.05; Table-II). After treatment, the NIHSS scores decreased significantly with respect to the baseline values in the three groups. The score after treatment in the combination group was lower than those in the aspirin and the clopidogrel groups (all *p*<0.05) (Table-III). After the treatment, the levels of serum inflammatory factors in the three groups were significantly lower than the baseline levels before treatment, and the combination group had the lowest level of all groups (all *p*<0.05) (Table-IV). After treatment, the levels of serum VILIP-1 and Cav-1 in the three groups were significantly lower than before the treatment, and the combination group level was the lowest of all (all *p*<0.05; Table-V).

**Table-II:** Comparison of treatment efficacy in the three groups [*n* (%)].

Group	n	Significant effective	Effective	Invalid	Total effective rate
Aspirin group	76	33 (43.42)	23 (30.26)	20 (26.32)	56 (73.68)
Clopidogrel group	72	36 (50.00)	21 (29.17)	15 (20.83)	57 (79.17)
Combination group	42	27 (64.29)	14 (33.33)	1 (2.38)	41 (97.62)^*#^
*χ*^2^Value	-	-	-	-	10.359
*p*-Value	-	-	-	-	0.006

* p<0.05 vs Aspirin group;

# p<0.05 vs Clopidogrel group.

**Table-III:** Comparison of NIHSS scores among the three groups (median (IQR)).

Group	n	Before treatment	After treatment	Z-Value	p-Value
Aspirin group	76	18 (18.19)	11 (10,13)	-7.593	<0.001
Clopidogrel group	72	18 (17,19)	10 (9,13)	-7.334	<0.001
Combination group	42	18.5 (17,19)	9.5 (9,11)^*#^	-5.600	<0.001
*H* value	-	1.688	16.528	-	-
p-Value	-	0.43	<0.001	-	-

* p<0.05 vs Aspirin group;

# p<0.05 vs Clopidogrel group.

**Table-IV:** Comparison of serum inflammatory factor levels among the three groups.

		IL-8 (ng/ml)		TNF-α (ng/L)		IL-1β (ng/L)	

Group	n	Before treatment	After treatment	Before treatment	After treatment	Before treatment	After treatment
Aspirin group	76	1.45±0.11	0.72±0.07^a^	73.70±7.25	35.86±3.48^a^	52.47±5.86	31.38±3.62^a^
Clopidogrel group	72	1.43±0.10	0.71±0.06^a^	73.01±6.12	34.55±4.08^a^	52.21±5.95	30.97±3.32^a^
Combination group	42	1.46±0.12	0.53±0.07^*#a^	73.26±5.93	25.84±2.67^*#a^	53.12±5.99	24.67±3.55^*#a^
F value	-	1.439	127.891	0.210	115.29	0.314	56.977
p-Value	-	0.240	<0.001	0.811	<0.001	0.731	<0.001

* p<0.05 vs Aspirin group;

# p<0.05 vs Clopidogrel group;

a p<0.05 vs baseline values.

**Table-V:** Comparison of serum VILIP-1 and Cav-1 levels among the three groups.

Group	n	VILIP-1 (pg/ml)		Cav-1 (ng/ml)	

		Before treatment	After treatment	Before treatment	After treatment
Aspirin group	76	642.62±44.08	585.58±40.67^a^	28.81±7.13	20.92±5.65^a^
Clopidogrel group	72	646.89±57.49	582.17±37.56^a^	28.48±6.30	20.93±4.61^a^
Combination group	42	642.82±58.93	513.85±26.64^*#a^	28.63±6.43	16.49±4.14^*#a^
*F-Value*	-	0.141	59.45	0.045	13.030
*p-Value*	-	0.868	<0.001	0.956	<0.001

* p<0.05 vs Aspirin group;

# p<0.05 vs Clopidogrel group;

a p<0.05 vs baseline values.

## DISCUSSION

Our results demonstrate that the patients receiving the combination regimen of aspirin and clopidogrel achieved the best outcomes of all those evaluated in our study. APCI is a multifactorial event that results from insufficient blood supply to the local brain tissues, causing ischemic and hypoxic damage to the brain that manifests as neurological deficits.^2^ Prompt interventions during the ischemic penumbra after the occurrence of cerebral infarction can reinstate the blood supply, thereby saving the otherwise dying nerve cells.^11^,^12^ Thrombolysis is the only widely recognized treatment to apply during the ischemic penumbra.^12^,^13^ However, thrombolytic therapy often has strict indications and treatment time windows that make it impractical in most situations.^13^ Thus, less restrictive treatments are needed.^11^-^13^

Clopidogrel is an antiplatelet drug that inhibits the binding of adenosine diphosphate (ADP) to platelet receptors competitively and thereby prevents platelet aggregation.^14^,^15^ Aspirin is a typical antiplatelet drug that regulates cyclooxygenase-1 activity pathways.^16^ The combination of these two drugs has been proposed to exert synergistic and complementary effects that should improve treatment efficacy.^11^-^15^ In this study, we found that the total clinical treatment efficacy increased after combined treatment, which is consistent with Prasad et al^6^, Yang et al.^7^, and Johnston et al^8^. Furthermore, the NIHSS scores of the patients in the combination group were the best in our whole study population after treatment, which is consistent with the results of Huang HY et al.^17^ and Shen et al.^18^ Aspirin regulates the acetylation of platelet cyclooxygenase, inhibiting the formation of serotonin A2 and inhibiting its involvement in platelet aggregation resulting in effective thrombolysis and anticoagulation. Clopidogrel can selectively block ADP receptors, it irreversibly binds to platelet receptors, inhibiting the binding of ADP to platelet receptors, effectively preventing platelet aggregation.^7^ In addition, clopidogrel bioavailability does not get affected by food or other factors after oral intake. The drug achieves a high blood concentration and takes effect quickly, helping to restore the blood supply to the brain, reducing neurological damage, and achieving effective disease control.^7^

In addition, we found that the levels of various serum inflammatory factors were lowest after the treatment with the combination regimen. This result is similar to those by Zhang et al. 19 showing that clopidogrel and aspirin can inhibit platelet aggregation in a variety of different ways, effectively inhibiting the expansion of thrombi, helping to alleviate the obstruction, and reducing the release of inflammatory factors from infarcted lesions. In addition, Ibrahim et al.^20^ found that clopidogrel can inhibit ADP-induced platelet activation, thereby preventing the aggregation of leukocytes and platelets with endothelial cells, and reducing the release of inflammatory cytokines.^19^,^20^

Furthermore, the levels of serum VILIP-1 and Cav-1 were also lowest in the patients receiving the combination therapy. VILIP-1 is a cytoplasmic protein widely expressed in brain neurons, where it mediates the calcium dependent signaling processes. Elevated levels of VILIP-1 can damage neural function.^21^ Cav-1 regulates nerve oxidative stresses and blood-brain barrier exudation. Increases in its level lead to the enlargement of edema zones around the infarcted site and aggravate the disease.^22^ Thus, the combination therapy may improve the efficacy of APCI treatments by reducing serum levels of VILIP-1 and Cav-1. However, further research is needed to elucidate the specific mechanisms of its actions and provide more evidence for future recommendations.

### Limitations:

This was a single center retrospective analysis, with a small sample size, and no long-term follow-up of patients; bias in the research results may have been unavoidable. Larger multicenter controlled trials should be conducted to validate our findings on the basis of more accurate and reliable data.

## CONCLUSION

Compared with aspirin alone or clopidogrel alone, a combination regimen of aspirin with clopidogrel is more effective for the treatment of APCI and can ameliorate the neurological impairment in patients. Our results also show that the combination regimen is more effective at decreasing the levels of serum inflammatory factors and of VILIP-1 and Cav-1. We believe these findings will be valuable for clinicians to improve patients’ outcomes.

### Authors’ contributions:

**HH:** Conceived and designed the study.

**SZ, HD, YG** and **HZ:** Collected the data and performed the analysis.

**HH:** Was involved in the writing of the manuscript and is responsible for the integrity of the study.

All authors have read and approved the final manuscript.
